# Astrocytes as targets for drug discovery

**DOI:** 10.1016/j.drudis.2018.01.011

**Published:** 2018-01-06

**Authors:** Kirill Gorshkov, Francis Aguisanda, Natasha Thorne, Wei Zheng

**Affiliations:** National Center for Advancing Translational Sciences, National Institutes of Health, Bethesda, MD 20892, USA

## Abstract

Recent studies have illuminated the crucial role of astrocytes in maintaining proper neuronal health and function. Abnormalities in astrocytic functions have now been implicated in the pathogenesis of neurodegenerative diseases, including Alzheimer’s disease (AD), Parkinson’s disease (PD), Huntington’s disease (HD), and amyotrophic lateral sclerosis (ALS). Historically, drug development programs for neurodegenerative diseases generally target only neurons, overlooking the contributions of astrocytes. Therefore, targeting both disease neurons and astrocytes offers a new approach for drug development for the treatment of neurological diseases. Looking forward, the co-culturing of human neurons with astrocytes could be the next evolutionary step in drug discovery for neurodegenerative diseases.

## Introduction

The search for neurodegenerative disease therapies has yielded only a few viable treatment solutions for patients. In the last 50 years, there have been no new major drugs for PD and no new therapeutic breakthroughs for AD. The complex etiology of these diseases requires innovative new thinking. Over the past 10 years, the push to understand the relationship between astrocytes and neurodegenerative disorders has set the stage for new therapeutic development opportunities to complement current drug development approaches that solely target disease neurons. Given the paucity of effective therapies despite the enormous investments in research and development, exploring patient-derived astrocytes as disease targets could uncover new primary, adjuvant, or combinatorial treatments for neurological diseases.

Astrocytes are non-neuronal cells that constitute a significant proportion of the central nervous system (CNS) cell population. Although the exact ratio of astrocytes to neurons is disputed [1], astrocytes have an essential role in maintaining neuronal health as well as the function of the blood–brain barrier (BBB). The morphological properties and spatial distribution of astrocytes contribute to CNS plasticity and the maintenance of neuronal homeostasis. A global analysis of glial fibrillary acidic protein (GFAP)-positive cells, (i.e., astrocytes) in the developing rat brain revealed large populations in the cortex and cerebellum with few cells in the brainstem throughout development, indicating that cell specification is regionally selective [2]. Concordantly, most research on diseases of the CNS has focused on the cortex and cerebellum because they are the sites of major degeneration.

When considering astrocytes for therapeutic development, particular attention should be paid to the heterogeneous nature of the astrocyte population [3]. They can be divided into three categories depending on their morphology and spatial distribution in the CNS. Protoplasmic astrocytes are located in the CNS gray matter, polarized astrocytes reside in the deep layers of the cortex, and fibrous astrocytes are found in the white matter [4]. Traditionally, they have been studied as a homogeneous group of cells. However, the patterning of neuronal stem cells (NSCs) in the developing brain generates unique subclasses of astrocytes. Research into the transcriptional profiles of astrocytes from different regions of the brain has determined that astrocytes display distinct molecular heterogeneity [5]. Although the functional consequences of these differences are not well established, understanding astrocytic subclasses is useful for guiding therapeutic development using astrocytes.

**FIGURE 1:**
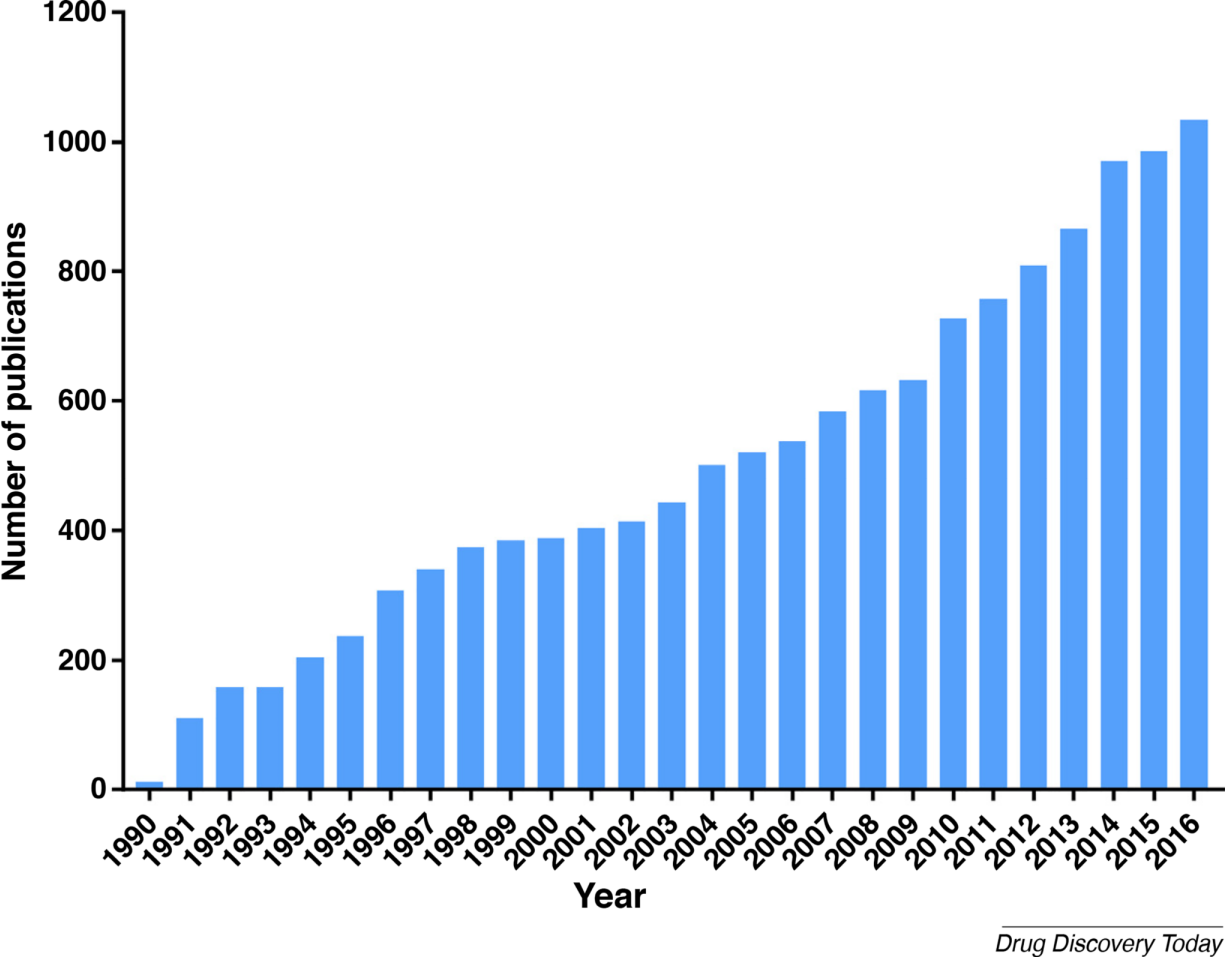
Number of publications per year for ‘astrocytes and disease’. An analysis of Web of Science search results for the search terms ‘astrocytes’ AND ‘disease’ revealed a steady increase in publications, including articles, reviews, and proceedings papers. Data from Web of Science (https://wcs.webofknowledge.com). Search completed in February 2017.

For many years, astrocytes received little to no consideration as targets for neurological drug discovery; neurons have too often been the sole focus for models used in developing new therapeutics. However, new findings expand the functional role of astrocytes in the CNS and show that they can induce or rescue neurons from disease states. Indeed, the number of research articles, reviews, and conference proceedings published on the topic of ‘astrocytes and disease’ has sharply increased, from 12 publications in 1990 to 1034 publications in 2016 (https://wcs.webofknowledge.com; Fig. 1). In this review, we describe current methods and technologies to generate and use astrocytes for disease modeling and emphasize the importance of targeting astrocytes in drug discovery and development for neurodegenerative disease therapies.

## Biological functions of astrocytes

One of the most-recognized roles of astrocytes is the homeostatic maintenance of neurotransmitters, such as glutamate and gamma-aminobutyric acid (GABA) [6]. Astrocytes have high-affinity transporters for these neurotransmitters and contain enzymes needed for their synthesis and metabolism. A lack of such regulation results in an increased propensity for neuronal glutamate excitotoxicity and subsequent cell death. Astrocytes also have transporters for K^+^, Na^+^/H^+^, HCO_3_^−^, and water, cementing their place as critical modulators of extracellular ion homeostasis [7].

Astrocytes also contact and extensively surround blood vessels in the brain and regulate local blood flow via the secretion of factors such as prostaglandins, nitric oxide, and arachidonic acid [8]. It is hypothesized that the dysregulation of vascular tone influences some disease pathologies, such as the inappropriate constriction of arterioles in response to aberrant calcium signaling in AD [9].

In addition to maintaining homeostasis for neurons, astrocytes also guide the formation and maturation of synapses [10]. Studies have shown that key astrocyte-secreted molecules, such as apolipoprotein E and thrombospondins, significantly promote synapse formation *in vitro* and *in vivo* [11]. Targeting astroglial control of synaptogenesis in neurodegenerative diseases could be a new strategy for therapeutic development. Astrocytes are also important for the regulation of reactive oxygen species (ROS), which harm the CNS by damaging DNA and increasing the oxidative burden. Large amounts of ROS are generated in the brain because of the high rates of oxidative metabolism. Astrocyte-derived glutathione, a key antioxidant, has been shown to be protective against neuronal cell death [12]. Interestingly, astrocytes themselves can generate harmful ROS when activated via immunological cytokines, such as interferon γ (IFN-γ) and interleukin 1β (IL-1β) [13]. Finally, astrocytes act as cellular sensors for hydrogen peroxide, which then drives the expression of hormones that influence the surrounding cellular microenvironment [14]. A summary of the functions of astrocytes in the CNS is provided in Table 1.

## Astrocytes and disease

Given the important role of astrocytes in supporting neuronal development and homeostasis, it is not surprising that their dysfunction, historically associated with loss-of-function phenotypes, contributes to a variety of neurodegenerative diseases. Recently, it was shown that diseases are also influenced by gain-of-toxicity changes within astrocytes. The early stages of many neurological diseases are characterized by inflammatory pathways that activate astrocytes, resulting in a process known as ‘reactive astrogliosis’, marked by increased levels of GFAP, further release of inflammatory cytokines, and morphological changes [15]. In some diseases, such as ALS, astrocytes can also secrete excess factors that are toxic to healthy neurons, such as transforming growth factor β1 (TGF-β1) [16]. An ALS study by Nagai *et al*. demonstrated that co-culture of Superoxide Dismutase 1 (SOD1) mutant astrocytes induced toxicity in healthy motor neurons (MN) [17].

Although activated astrocytes have been shown to migrate in scratch wound assays *in vitro* [18], active migration did not occur in a stab-wound mouse model of traumatic brain injury (TBI), but instead induced astrocyte proliferation [19]. Therefore, the astrocytic response to injury is likely to be physiologically context dependent, including the type of injury, location in the CNS, and other factors. Nonetheless, reactive astrogliosis during acute or chronic insult can cause glial scarring, a phenomenon that isolates the injured area while potentially preventing cellular repair and recovery [20]. However, this view is challenged by a recent report that suggested that astrocytic scars aid neuronal regrowth [21]. Given these conflicting reports, the field of astrocyte research in disease requires more efforts to better frame the complexities in astrocyte–neuron interactions. Examples of astrocyte involvement in disease pathogenesis or neuroprotection are detailed in Table 2.

## Examples of astrocytes affecting neurons in specific diseases

### Alzheimer’s disease

The importance of astrocytes as therapeutic targets in neurodegeneration is evident in AD, where large accumulations of tau proteins in astrocytes have been associated with reduced expression of astrocyte glutamate transporters [22]. Astrocytes are able to release both pro- and anti-inflammatory cytokines depending on the environmental context and, in the case of AD, astrocytes are proinflammatory and lead to increased cell death. The molecular mechanism for this was determined by Garwood *et al*., who used exogenous amyloid β (Aβ) oligomers to model AD in primary cortical neurons co-cultured with astrocytes [23]. The authors found upregulated inflammatory cytokines (IL-β, IL-6, and IFN-γ) that led to increased caspase-3 activity, cleavage of toxic tau, and neuronal cell death. When the astrocytic inflammatory response was attenuated by minocycline, neurons exhibited less Aβ-induced toxicity. In another study, AD astrocytes were found to also secrete α1-anti-chymotrypsin, which is toxic to neurons [24]. Therefore, targeting astrocytic cytokine release in an effort to reduce Aβ toxicity could be a viable strategy to alleviate AD pathology in humans.

### Parkinson’s disease

One form of PD, DJ-1 Parkinsonism, is caused by a mutation of a single gene. To understand the etiology of this disease, Mullett *et al*. co-cultured mutant astrocytes and dopaminergic neurons to assess astrocyte-mediated neuroprotection and found that astrocytes with deficient DJ-1 were unable to protect dopaminergic neurons from oxidative stress [25]. When cells were treated with mitochondrial complex I inhibitors to simulate oxidative stress, the dysfunctional astrocytes could not prevent dopaminergic cell death. Therefore, the authors concluded that DJ-1 mediates neuroprotection through an astrocyte-dependent mechanism involving extracellular-secreted soluble factors. This research exemplifies why it is important to study cellular function with physiologically relevant models where multiple cell types are integrated in a single biological context. Without the co-culture of astrocytes and neurons, it would not be possible to establish the mechanism of action of DJ-1 deficiency and the important role of astrocytes in protecting neurons susceptible to cell death in this form of PD.

### Huntington’s disease

In HD astrocytes, large accumulations of tau proteins are associated with the reduced expression of glutamate transporters, a phenotype also observed in HD neurons [26]. In addition to glutamate transporters, the loss of functioning ion channels can contribute to disease pathology, as is the case for a HD mouse model where reduced expression of Kir4.1 potassium channels leads to neuronal dysfunction [27]. Furthermore, in a well-established HD mouse model, cell signaling mediated by increased expression of GSK-3β in astrocytes led to increased proinflammatory cytokine release and was the main driver of increased tau hyperphosphorylation and caspase-3-mediated neuronal cell death [28]. The researchers conducted experiments with co-culture combinations of wild-type and diseased neurons and astrocytes to understand the contribution of astrocyte function to neuronal health in HD. When diseased astrocytes were layered on top of wild-type neurons, or vice versa, neuronal viability decreased. Furthermore, when GSK-3β was depleted in diseased astrocytes, tau phosphorylation and caspase-3 activation decreased and neuronal viability increased. The data provide evidence for a pathological interaction between astrocytes and neurons that might be involved in HD pathogenesis. Therefore, co-culture of astrocytes and neurons can provide mechanistic insights into the loss- and gain-of-function effects beyond phenotypic analysis. In addition, the co-culture of disease astrocytes and disease neurons can be a more-appropriate cell-based disease model for drug screening and for evaluation of drug efficacy.

### Rett syndrome

When modeling diseases of the CNS, it is now clear that the interactions between astrocytes and their neighboring neurons cannot be ignored. The co-culture of diseased astrocytes and wild-type neurons can elucidate the interactions between these two cell types and the role of astrocytes in neurodegeneration or inflammation. To illustrate this, Williams *et al*. generated astrocytes from patients with Rett syndrome (RTT) and observed their effects on wild-type neurons [29]. When co-cultured with RTT astrocytes, healthy neurons exhibited reductions in soma size, neurite length, and number of terminal ends. Interestingly, when wild-type neurons were cultured in media preconditioned by RTT astrocytes, these neurons again showed smaller soma size, shorter neurite length, and fewer terminal ends. This suggests the presence or absence of a secreted factor that regulates these changes in growth in wild-type neurons.

## Targeting astrocytes for therapeutic development

Astrocytes are uniquely positioned to promote the regeneration of damaged nerve cells or protect existing cells from degeneration in the CNS [30,31]. Two clinically relevant strategies that take advantage of these properties are astrocyte transplantation and pharmacological correction of astrocyte dysfunction. With respect to the former, disease-relevant human astrocytes can be acquired from primary sources or from differentiated embryonic stem cells (ESCs) or induced pluripotent stem cells (iPSCs). Investigators interested in using primary astrocytes from tissue can obtain them from commercial sources. Robust protocols for enriching primary astrocytes yield a cell population that is >98% pure [32]. However, because astrocytes are not a highly proliferative cell type, obtaining the large numbers of primary cells necessary for drug discovery campaigns is a major challenge.

### Astrocytes from induced pluripotent stem cells

Pluripotent cells can be induced from somatic cells (generally fibroblasts or blood mononuclear cells) that are subsequently differentiated into cell types of interest, such as neurons, cardiomyocytes, and hepatocytes [33]. In addition to providing researchers with large numbers of cells for assay development and screening, iPSCs have the added benefit of producing cells with the original patient-specific genetics. Over the past 4 years, several labs have generated both ESC- and iPSC-derived astrocytes [5,34–36]. Although the astrocytes differentiated from iPSC sources can produce a large number of astrocytes, the current methods for astrocyte differentiation have not yet been fully developed and need further improvement and optimization. Additionally, the field is in need of protocol standards for the generation and characterization of mature astrocyte populations. Alternatively, expedited protocols circumvent the need for dedifferentiation to the iPSC stage and convert fibroblasts directly into astrocytes [34], although the purity of astrocytes using this method is low. The maturation of stem cell differentiation techniques is critical for efficiently generating patient-derived astrocytes for use in investigating the molecular mechanisms of disease and performing com-pound screening. Additionally, *in vitro* gene editing of iPSCs and astrocytes from patients can be used for autologous transplantation in the treatment of disease [37].

### Using human induced pluripotent stem cell-derived neurons and astrocytes

Once healthy astrocytes have been obtained and characterized, they can be transplanted to replace dying astrocytes or to promote the survival of existing neurons. Although these strategies have not yet been tested in humans, there is a growing body of *in vitro* and *in vivo* evidence that suggests these therapies would be beneficial for many neuronal diseases. In an ALS mouse model, researchers have shown that the direct transplantation of human (h)iPSC-derived neural progenitor cells (NPCs) extended the life-span of the animal [38]. In these experiments, NPCs differentiated into astrocytes and exhibited an upregulation of vascular endothelial growth factor (VEGF), which increased AKT signaling, which has previously been shown to be important for cell survival in ALS [39]. The authors also hypothesized that the introduction of progenitor-derived astrocytes with normal expression of glucose transporters could restore glucose homeostasis in this model. The hiPSC derivation and co-culture of neuronal subsets and astrocytes can reveal both cell-autonomous and noncell-autonomous effects of disease on different cell types in a single system. Using ALS as an example, Hall *et al*. demonstrated how a diminished support capacity of patient-specific valosin-containing protein (VCP) mutant astrocytes led to a decrease in cell viability in control MNs compared with co-culture with control astrocytes [40]. Additionally, in this study, VCP MNs exhibited lower survival when cultured with VCP astrocytes compared with control astrocytes. Thus, incorporating astrocytes into neuronal studies, facilitated by the development and use of hiPSC technology, can lead to molecular insights of disease pathogenesis and enhance new therapeutic discovery. For example, Ishi *et al*. utilized hiPSC-derived astrocytes and neurons together to study drug-induced seizures, a model required for therapeutic risk assessment. The researchers revealed that only co-culture of hiPSC-derived astrocytes facilitated the maturation of hiPSC-derived neurons and allowed for the detection of drug-induced seizure activity [41]. These experiments demonstrate how the interaction between disease neurons and astrocytes in co-culture can serve as a better phenotypic disease model for drug screening.

Although astrocyte transplantation could be a viable strategy for replacing dying neurons, it does not directly address a patient’s endogenous dysfunctional astrocytes. For example, restoring glutamate homeostasis in AD astrocytes or reducing toxic cytokine production in ALS astrocytes, in addition to restoring neuronal function, is critical to a holistic approach in treating these diseases. One strategy is to conduct drug-screening campaigns with diseased cell populations of astrocytes and neurons to identify effective small-molecule therapeutics that will abolish the disease phenotype. The success of these compound-screening campaigns is dependent on the use of appropriate cell-based disease models. Given the high proliferative capacity of iPSCs, researchers can use the cell-based disease models to conduct large-scale drug screens once proper assays are developed. Additionally, these models allow for further dissection of the biological roles of astrocytes in neurodegenerative diseases from patients with varying genetic backgrounds. Many groups have used patient-derived iPSCs to generate astrocytes and model neurodegenerative disease *in vitro* and a summary of such iPSC-derived human astrocyte models is provided in Table 3.

## Drug-screening strategies using astrocytes

The recent availability of iPSC-derived astrocyte disease models enables high-throughput drug-screening campaigns to utilize a disease-relevant phenotypical approach for therapeutic discovery. Molecular target-based screening, one major strategy for drug discovery, has been popular in the pharmaceutical industry for the past 25 years [42]. Target-based screening is dependent on target identification relevant to the disease of interest, such as potassium ion channels in HD astrocytes or glutamate receptors in PD astrocytes. Thanks to decades of basic science research examining the mechanisms of astrocyte function, there are numerous potential astrocytic targets for assay development. However, target-based screens might limit the possibility of identifying novel mechanisms of action or might not move forward readily if the pathogenesis of a disease is unclear.

### Phenotypic assays using astrocytes

In contrast to target-based screening, phenotypic assays focus on cellular pathological characteristics that can be modulated by a bioactive compound. For astrocytes in neurodegenerative diseases, compounds can be evaluated for efficacy in preventing oxidative stress-related cell death or as a measure of cell health, which can be measured by intracellular glucose concentrations. Indeed, the disruption of proper glucose reuptake has been shown to precede Aβ plaque accumulation in patients with AD [43]. Established assays that monitor glucose uptake can be used to evaluate the ability of a compound to restore glucose homeostasis [44]. Using a co-culture system, compounds can be evaluated for their ability to prevent neuronal death by ameliorating disease phenotypes in patient astrocytes. To differentiate the effects on astrocytes versus neurons during high-content screening, co-cultures can be immunostained with GFAP for astrocytes and neural-specific tubulin for neurons. Common measurements include neuron cell count, neurite length, astrocyte cell count, astrocyte area, and GFAP expression. Phenotypic screens have the potential to be more physiologically relevant and could take advantage of polypharmacology by uncovering compounds that interact with multiple targets. Target-based screens have routinely yielded drug candidates that later fail in clinical trials for diseases such as AD and PD. Phenotypic screens of co-cultured patient-derived astrocytes and neurons could yield novel lead compounds and drug targets, due, in part, to the unbiased nature of phenotypic screens.

### High-throughput screening for neurotoxicity using iPSC technology

In addition to investigating disease, healthy astrocytes can be used to evaluate compounds for neurotoxicity or the ability of these compounds to protect astrocytes from disease. In one such experiment, Pei *et al*. used isogenic iPSCs, NSCs, iPSC-derived astrocytes, and iPSC-derived neurons to test the cytotoxicity of 80 known neurotoxins and environmental compounds [45]. Out of the 80 compounds tested, 41 were shown to be cytotoxic to astrocytes, as measured by the 3-(4,5-dimethylthiazol-2-yl)-2,5-diphenyltetra-zolium bromide) tetrazolium (MTT) cell viability assay. Interestingly, for compounds that were toxic to all four cell types, astrocytes exhibited the least cytotoxicity. Human iPSC-derived astrocytes and other cell lines are a powerful platform for evaluating drug toxicity without relying on animal models [46]. Although these experiments were performed in a 96-well plate format that served as a proof of concept, scaling the assay into 384- and 1536-well plate formats will enable rapid screening of large amounts of known environmental chemicals and compounds for neurotoxicity. Furthermore, expanding on the complexity of the culture by using a 3D co-culture of human neurons and astrocytes increases the physiological relevance of such screening systems, given the ubiquitous colocalization of astrocytes and neurons in the brain [47].

Stem cell-derived astrocytes could also be used to find compounds that protect them from insults within the CNS, such as ROS. For example, hESC-derived astrocytes were used to develop a phenotypic assay for compounds that protect against oxidative stress. Oxidative damage is associated with the pathologies of many neurological diseases and, thus, the identification of compounds that protect against this stress could halt or significantly slow disease progression. Approximately 4100 bioactive compounds were screened in a 1536-well plate format for their ability to protect astrocytes from hydrogen peroxide-induced oxidative damage using nuclear morphology as a protection indicator [48]. Twenty-two of the approximately 4100 compounds elicited a protective nuclear profile in astrocytes. This study pioneered high-throughput screening using differentiated human astrocytes and is the first example of using iPSC-derived astrocytes to screen for compounds in a high-density 1536-well plate format. The generation of the 1.4 × 10^8^ cells necessary for this type of screen required optimization of the stem-cell differentiation protocol.

## Concluding remarks and future directions

Astrocytes represent an important new focus in therapeutic development for disorders that have been historically viewed as purely neuronal in their pathology. Within the past 5 years, important progress has been made deriving astrocytes from iPSCs. Researchers are now able to generate patient-specific astrocytes that accurately recapitulate a patient’s genetic background and disease phenotype. These versatile human astrocytes could be used by themselves or co-cultured with neurons in both target-based and phenotypic high-throughput drug-screening campaigns, promoting the discovery of novel therapeutics to treat neurological diseases.

Conducting drug-screening campaigns using both patient-derived neurons and astrocytes paves the way for a phenotypic and polypharmacological approach to drug discovery. For decades, therapeutic development for neurodegenerative disorders has focused on addressing neuronal manifestations by only targeting disease neurons. Given the critical role of astrocytes in pathogenesis, it is not surprising that the traditional drug development strategy of solely targeting diseased neurons has not produced the therapies that patients desperately need. Developing new drugs with polypharmacological activities or for drug combination therapies that target both neuronal and astrocytic degeneration could provide a new direction to increase the success of therapeutic development for neurodegenerative diseases in the future.

The shift from a neuron-centric view to one that incorporates astrocytes in disease models for drug discovery is a critical step in renewing drug development campaigns to treat neurodegenerative diseases. For AD and dementia, it is estimated that the cost of care will be in excess of US$1.1 trillion in the USA by 2050 [49]. Therefore, it is paramount that we develop drug therapies efficiently by using human neuronal cells and disease-relevant model systems. Human stem cell-derived astrocytes in combination with neurons are a powerful platform both for investigating the basic biology of disease as well as finding novel therapeutics. Tapping into the power of astrocyte biology by including them in drug screens for neurological disorders will lead to the discovery of drugs desperately needed by patients with these diseases.

## Figures and Tables

**TABLE 1:** Biological functions of astrocytes

Role	Biological process	Refs
CNS development	Astrocytes develop after neurons; guide migration of neural progenitors; facilitate synapse formation and pruning; maintain myelin	[10]
Barrier functions	At the BBB, astrocytic endfeet serve to induce barrier properties in cerebral and endothelial cells	[4,7]
Homeostasis	Astrocytes have specialized channels to regulate fluids; channel neurotransmitters, such as glutamate and GABA; small-molecule regulation through gap junctions	[6]
Metabolic support	Glucose regulation is a major function of astrocytes in the CNS; astrocytic glycogen helps support neurons when concentrations of glucose are low	[4]
Synaptic transmission	Astrocytes release molecules to activate synapses directly, and can modulate neuronal excitability through their own Ca^2+^ concentrations; growth factors can regulate synaptic transmission over longer time periods	[10]
Neurovascular support	Astrocytes contact blood vessels; release second messengers, such as nitric oxide, prostaglandins, and arachidonic acid; regulate changes in blood flow in response to electrophysiological activity	[8,9,50]

**TABLE 2:** Influence of astrocytes on neurodegenerative disease pathogenesis

Disease	Cellular presentation	Clinical presentation	Prognosis	Possible roles of astrocytes in pathogenesis/neuroprotection	Refs
AD	Accumulation of two protein aggregates, Aβ plaques and tau neurofibrillary tangles	Early and significant memory impairments	Variable. Most cases present after 65 years of age	Activated astrocytes overexpress α1-anti-chymotrypsin, a known inhibitor of Aβ degradation; increase in influx of GSH, hydrogen peroxide, and lactate; impaired glucose homeostasis; reduction in glutamate transporters	[24,51]
PD	Loss of dopaminergic neurons, presence of Lewy bodies	Tremors, stiffness, postural instability, and slowness of movement	Disease itself is not fatal	Production of neurotoxic species, such as glutamate, S100B, TNF-α, ROS, and nitrogen species; reduction in normal levels of heat shock protein 70 (Hsp-70) and antiapoptotic proteins	[52–54]
HD	Expanded trinucleotide repeat (CAG) in huntingtin gene correlates with disease severity; unclear how mutation directly causes the disease	Dementia, unwanted choreatic movements, psychiatric disturbances	15–20 years from onset	Impaired glutamate uptake; mutant huntingtin causes reduced levels of cholesterol biosynthesis in astrocytes; abnormal potassium ion channel function; wild-type astrocyte-conditioned media rescues synaptogenesis in Huntington neurons	[26,27,55]
RTT	*De novo* genetic mutation of *MECP2* gene on X chromosome	Female developmental disorder in early childhood; motor issues; seizures, scoliosis; sleeping problems	Males die within first 2 years of birth, females live up to 40 years or more	RTT astrocytes can spread MeCP2 to neurons and cause abnormal neurodevelopment	[56,57]
ALS	Heterogeneous; 10% of cases due to known familial mutations; 90% sporadic	Muscle atrophy and weakness because of motor neuron degeneration	3–5 years after diagnosis	SOD1 mutant astrocytes are toxic to healthy motor neurons via astrocyte-derived TGF-β1; healthy astrocytes are neuroprotective to SOD1-mutant motor neurons, because of their normal glutamate receptors	[17,58,59]

**TABLE 3:** Currently established iPSC-derived human astrocyte models of neurodegenerative diseases

Disease	iPSC generation method	Astrocyte differentiation method	Cellular phenotypes	Refs
AD	Episomal	Exposure of neural progenitors to N2 for 8 weeks	Accumulation of Aβ oligomers, Increased ROS	[60]
HD	Retrovirus	Exposure of neural progenitors to astrocyte medium (ScienCell) for 2–3 months	High numbers of cytoplasmic vacuoles, correlating with CAG repeat length	[61]
TDP-43 neuropathy (ALS)	Retrovirus	Neural precursor cells cultured in medium containing EGF and LIF for 4–6 weeks	TDP-43 inclusions; increased cell death	[62]
Down’s syndrome	Sendai virus	Neurosphere BMP4/FGF-2 exposure for 20 days	Higher levels of GFAP and S100B; higher nitric oxide generation; impaired levels of NFE2L2, a mediator of GSH production; impaired levels of TSP-1 and TSP-2, critical factors for synapse formation	[63]
RTT	Lentivirus	Neural precursor cells grown in EGF and FGF2 for 90–300 days	Reduced soma size, neurite length, and terminal ends in co-cultured healthy neurons; responsive to therapeutics currently in clinical trials for RTT	[29]
